# Uncertainty weighted multi task learning for robust traffic scene semantic understanding

**DOI:** 10.1038/s41598-025-24838-3

**Published:** 2025-11-20

**Authors:** Zhiping Wan, Shitong Ye, Feng Wang, Shaojiang Liu, Ling Peng

**Affiliations:** 1 School of Information and Intelligence Engineering, Guangzhou Xinhua University, Dongguan, 523133 China; 2 School of Artificial Intelligence, Guangzhou Huashang College, Guangzhou, 511300 China; 3 School of Artificial Intelligence, Dongguan City University, Dongguan, 523419 China

**Keywords:** Uncertainty weighting, Multi-task learning, BEV representation, Mixture of experts transformer, Semantic understanding of traffic scenes, Engineering, Mathematics and computing

## Abstract

This paper addresses perception degradation caused by adverse weather, occlusion, and asynchronous sampling by proposing an uncertainty-weighted multi-task learning framework for robust semantic understanding of traffic scenes (UW-MTL). The method performs differentiable multi-source spatiotemporal alignment to unify camera, LiDAR, radar, and IMU into a BEV sequence, and adopts a hybrid backbone that combines a Mixture of Experts Transformer with a spatiotemporal graph neural network to balance global semantics and local topology. Each task employs evidential prediction heads that explicitly output confidence and uncertainty. During training, soft-temperature weighting and a sigma aware gradient conflict resolver enable stable joint optimization. On the nuScenes benchmark, UW-MTL consistently surpasses BEVFusion and UniAD on 3D object detection, BEV semantic segmentation, and short-horizon trajectory prediction, with especially pronounced gains at long range, under heavy occlusion, and in low-visibility conditions.

## Introduction

Intelligent transportation is transitioning from a human-driven paradigm to a software-defined system centered on perception and decision-making. To operate safely on real urban roads, perception modules must not only identify vehicles, pedestrians, cyclists, and dynamic obstacles, but also understand road semantics under varying weather and lighting conditions, including drivable areas, lane markings, median strips, intersection topology, and the resulting traffic constraints^1,2^. Unlike controlled environments, urban roads are subject to adverse factors such as rain, fog, strong reflections, low illumination at night, high-speed vehicle intersections, long-tail vehicle shapes, and heavy occlusions. Sensors themselves also suffer from measurement noise and field-of-view limitations^3,4^. Single-modal approaches are prone to failure in such scenarios, making multi-modal fusion the mainstream trend. To enhance efficiency and global consistency, an increasing number of studies are integrating detection, segmentation, depth estimation, and trajectory prediction into a multi-task learning framework within the feature space of a bird’s-eye view^5–7^, aiming to capture the semantic and geometric characteristics of traffic scenes in a shared representation.

Currently, multi-task learning methods for transportation scenarios still face three fundamental challenges^8–10^. The first challenge is the contradiction between the differentiability and accuracy of cross-modal spatio-temporal alignment. The sampling frequency, exposure delay, and external parameter errors of cameras, lidars, millimeter-wave radars, and inertial navigation systems can all lead to coordinate mismatches. If the alignment is not differentiable, end-to-end training will be difficult to correct. If the alignment is coarse, the fusion effect will be limited by artifacts. The second challenge is gradient conflicts and resource competition among multi-tasks. Detection prioritizes the consistency of object contours and scales, segmentation emphasizes the fineness of regional boundaries, depth estimation relies on geometric priors, and trajectory prediction requires temporal continuity and interactive modeling. These objectives often exhibit conflicting optimization directions on a shared backbone, leading to negative transfer. The third category involves the measurement and utilization of uncertainty. Observational uncertainty stems from sensor noise and occlusion, while model uncertainty is related to data coverage and capacity. If uncertainty is not incorporated into optimization and routing, it is difficult to suppress noise dominance during training and to provide risk-aware confidence for planning and control during inference.

The current unified BEV representation demonstrates advantages in terms of geometric consistency and convenience for downstream tasks, but it typically requires a trade-off between precise spatio-temporal alignment and end-to-end trainability. Multi-task learning offers significant benefits in terms of parameter sharing and efficiency, but fixed weights or heuristic weights often become unstable in noisy scenarios and may even amplify the impact of difficult samples. Research on uncertainty has primarily focused on evaluation or visualization stages, with limited exploration of incorporating confidence as a core signal for training and computational resource scheduling throughout the entire process. In terms of architectural design, Transformers excel at modeling long-range dependencies, while graph neural networks can capture local topology and interactions. However, there remains a lack of unified and efficient practices for stably integrating these two approaches within the BEV space.

Based on the above background, this paper proposes a unified multi-task paradigm built around uncertainty. The core idea is to elevate uncertainty from ancillary information to first-class citizenship, enabling it to participate simultaneously in data alignment, feature routing, loss balancing, and gradient analysis. This suppresses noise dominance in shared representations, alleviates task conflicts, and allocates more effective computation to difficult regions. After differentiable multi-source alignment, this paper unifies multi-modal observations into a BEV temporal grid and explicitly encodes registration residuals to expose noise sources. In the representation learning stage, a hybrid backbone composed of a Mixture of Experts Transformer and a spatio-temporal graph neural network is employed to capture global context and local topology, respectively. At the task level, evidence-based learning is used to predict outputs and uncertainty for discrete and continuous tasks. At the optimization level, soft temperature uncertainty weighting is utilized for adaptive task balancing, and priority-based gradient conflict resolution is employed to reduce negative transfer, while the latest uncertainty is fed back to expert routing.

The main contributions of this paper are as follows:

(1) We propose a differentiable multi-source alignment and BEV unified representation for semantic understanding, which jointly encodes appearance, geometry, motion, and registration residuals to provide robust inputs for multi-task training.

(2) Designing a hybrid backbone that combines a Mixture of Experts-based Transformer with a spatio-temporal graph neural network to uniformly model long-range dependencies and local topological relationships in BEV space.

(3) Adopting an evidence-based task head to simultaneously provide predictions and uncertainty in a single forward pass, and achieving adaptive task balancing across training stages through soft temperature weighting.

The structure of this paper is as follows: Sect. 2 reviews previous work related to this study. Section 3 defines the problem and introduces the notation system, describing the construction of multi-source input representations that are differentiably aligned to the BEV in urban traffic scenarios, and formalizing the joint learning objective and uncertainty characterization. Section 4 provides a detailed explanation of the proposed framework, including key modules such as multi-source spatio-temporal alignment, a hybrid backbone composed of MoE and spatio-temporal graph neural networks, an evidence-based task head, uncertainty weighting, and gradient conflict resolution. Section 5 reports on the experimental setup, evaluation metrics, and comparison results, verifying the consistent benefits and efficiency of the method across multiple tasks. Section 6 summarizes the entire paper and discusses the applicability boundaries of the current method and potential directions for improvement.

## Related work

In recent years, to achieve robust traffic scene understanding, the academic community has proposed various methods combining multimodal perception and multi-task learning. Early strategies such as PointPainting^11^ employed point-by-point fusion, attaching semantic labels from image segmentation to lidar point clouds to enhance detection performance, but were susceptible to calibration errors and degraded image quality. To address these issues, BEVFusion^12^ fuses multimodal features in a shared bird’s-eye view (BEV) space, effectively preserving the semantic density of images and the geometric accuracy of lidar, significantly improving 3D detection and BEV segmentation accuracy on nuScenes. TransFusion^13^ utilizes Transformers to fuse multimodal features and enhances robustness against distortion and parallax errors through soft alignment.

In terms of pure visual perception, multi-view BEV representations have achieved significant breakthroughs. Lift-Splat-Shoot (LSS)^14^ proposed an approach to implicitly generate 3D voxels from multi-camera images and project them onto the BEV plane, achieving end-to-end learning for the first time. Subsequently, BEVFormer^15^ learned a unified BEV representation across multiple time steps and viewpoints using a spatio-temporal Transformer encoder, and enhanced temporal consistency through cross-attention mechanisms, achieving a NDS of 56.9% on nuScenes, with performance approaching that of lidar-based methods. As BEV representations gain widespread adoption, integrated multi-task perception frameworks are attracting increasing attention. BEVerse^16^ jointly performs detection, segmentation, and trajectory prediction on the spatio-temporal BEV representation, with multi-task collaboration leading to overall performance improvements. UniAD^17^ takes this further by extending multi-task learning to the entire autonomous driving pipeline, simultaneously modeling perception, prediction, and planning tasks within a unified query-based interaction framework. It sets new records for multiple tasks on the nuScenes dataset, highlighting the advantages of a planning-oriented end-to-end design.

To model the complex relationships between traffic participants and road structures, graph neural networks (GNNs) have been introduced into scene understanding. VectorNet^18^ represents map elements and trajectories as vector sequences and uses hierarchical GNNs to extract local and global relationships, significantly outperforming grid-based methods in prediction tasks. LaneGCN^19^ directly performs graph convolution and attention mechanisms on the lane centerline topological graph, capturing lane, vehicle, and their interaction dependencies, thereby improving the accuracy of multimodal trajectory prediction. On the other hand, uncertainty modeling has become a key means of improving robustness. Kendall et al.^20^ proposed a multi-task loss based on uncertainty dynamic weighting, which adaptively balances task learning without the need for manual parameter tuning. Sensoy et al.^21^ proposed an evidence-based learning method based on the Dirichlet distribution, which provides confidence estimates for classification predictions and enhances robustness to outlier samples.

Compared to existing methods, this paper constructs a differentiable alignment chain from sensors to BEV and explicitly writes the registration residual into the channel as a priori clue for subsequent uncertainty and robust semantic fusion. Compared to schemes that only perform static BEV fusion or single routing, this paper provides more calibrated confidence and stronger robustness in long-range, heavy occlusion, low visibility, and high-speed conditions while maintaining controllable latency and parameter scale.

## Problem definition

### Scene and input representation

In highly dynamic urban road environments with complex perception conditions, such as rain, fog, nighttime, strong light, and obstructions, a single sensor often fails due to limited visibility or amplified noise, thereby weakening the robustness of downstream scene understanding. To address this, this paper jointly collects four types of heterogeneous modalities within a continuous time window $\:[t-T,t]$, RGB images $\:{\mathbf{I}}^{\text{cam}}\in\:{\mathbb{R}}^{{H}_{c}\times\:{W}_{c}\times\:3}$ provide high-resolution texture and semantic information, LiDAR point clouds $\:{\mathbf{P}}^{\text{lidar}}\subset\:{\mathbb{R}}^{3}$ provide centimeter-level 3D geometry, millimeter-wave radar echoes $\:{\mathbf{R}}^{\text{radar}}\subset\:{\mathbb{R}}^{4}$ supplement all-weather motion information, and IMU six-axis attitude $\:{\mathbf{u}}^{\text{imu}}\in\:{\mathbb{R}}^{6}$ provides a high-frequency pose reference for temporal alignment of multi-source data.

To unify semantic, geometric, and motion information at the representation level and suppress cross-modal spatio-temporal biases, a differentiable coordinate consistency mapping $\:M(\cdot\:;{{\Theta\:}}_{M})$ is constructed to aggregate multi-source observations into a semantically friendly BEV space. All 3D measurements are normalized to the vehicle coordinate system via the calibration matrix $\:{T}_{V}^{S}\in\:SE\left(3\right)$, then written into the BEV grid by the projection operator $\:{{\Pi\:}}_{\text{B}\text{E}\text{V}}$. Camera pixels are combined with depth priors and back-projected into BEV coordinates aligned with LiDAR/Radar. Their spatial writing and gridification use continuous differentiable soft-voxelization to preserve end-to-end semantic learnability:

1 $$\:v={{\Pi\:}}_{\text{B}\text{E}\text{V}}\left({T}_{V}^{S}{p}^{S}\right)$$

2 $$\:{Z}_{t}(x,y)=\sum\:_{i}{\omega\:}_{i}{\varphi\:}_{i}(x,y)$$

3 $$\:{\omega\:}_{i}=max(\text{0,1}-\Vert\:{v}_{i}-(x,y){\Vert\:}_{1}/\delta\:)$$

Where $\:{\varphi\:}_{i}(x,y)$ represents semantic feature fragments from different modalities, including local contributions from appearance embeddings, geometric descriptions, and motion cues. $\:{\omega\:}_{i}$ ensures a continuous, differentiable mapping from sparse to dense, enabling smooth spatial fusion of semantic evidence and reducing category fragmentation caused by discretization. In the temporal dimension, the continuous pose $\:{T}_{t}^{{t}^{{\prime\:}}}$ provided by the IMU is used to back-transform asynchronous LiDAR/Radar frames to the reference time *t*, and spline interpolation is performed on low-frame-rate modalities to fill in temporal density. The residuals from interpolation and registration are encoded as additional channels and input alongside $\:{Z}_{t}$ to explicitly expose the sources of semantic uncertainty. The final result is a serialized BEV semantic tensor:

4 $$\:{Z}_{t}=M({I}_{t}^{\text{c}\text{a}\text{m}},{P}_{t}^{\text{l}\text{i}\text{d}\text{a}\text{r}},{R}_{t}^{\text{r}\text{a}\text{d}\text{a}\text{r}},{u}_{t}^{\text{i}\text{m}\text{u}})\in\:{\mathbb{R}}^{H\times\:W\times\:C}$$

The channel *C* is composed of appearance, geometry, motion, and alignment residual signals, and further provides contextual support for semantic stability and long-term context modeling by obtaining the continuous time series $\:\{{Z}_{t-T},\dots\:,{Z}_{t}\}$.

### Joint learning objectives

While ensuring automotive-grade real-time performance, the network must simultaneously perform four core tasks on the aligned BEV sequence: 3D object detection $\:{\mathbf{S}}_{t}^{\text{det}}$, semantic segmentation $\:{\mathbf{M}}_{t}^{\text{seg}}$, monocular depth estimation $\:{\mathbf{D}}_{t}^{\text{depth}}$, and short-term trajectory prediction $\:{\mathbf{Q}}_{t}^{\text{traj}}$. These four tasks cover both spatial entities—including real-time understanding of objects, semantics, and depth—and temporal behavior (trajectories), forming the minimal complete combination required for robust traffic scene understanding. To avoid performance degradation caused by single-task bias, this paper performs end-to-end joint optimization of the task set $\:\mathcal{T}=\{\text{d}\text{e}\text{t},\text{s}\text{e}\text{g},\text{d}\text{e}\text{p}\text{t}\text{h},\text{t}\text{r}\text{a}\text{j}\}$ within a unified feature space, and explicitly quantifies the uncertainty of each task to guide loss balancing and gradient scheduling.

Classification tasks, including detection and segmentation, use Dirichlet evidence modeling, and the network outputs a concentration vector $\:{\varvec{\alpha\:}}_{k}$, whose uncertainty is simplified to:

5 $$\:{\sigma\:}_{k}^{2}=1/({\alpha\:}_{k}-1)$$

For regression tasks involving depth and trajectory, the Normal–Inverse–Gamma (NIG) distribution parameters $\:({\mu\:}_{k},{\lambda\:}_{k},{\alpha\:}_{k},{\beta\:}_{k})$ are used, with the overall variance being:

6 $$\:{\sigma\:}_{k}^{2}={\beta\:}_{k}\left[{\lambda\:}_{k}\right({\alpha\:}_{k}-1){]}^{-1}$$

The obtained $\:{\sigma\:}_{k}^{2}$ will be used for task adaptive weighting and gradient conflict determination. Let the native loss of each task be $\:{\mathcal{L}}_{k}$. Based on uncertainty, a soft temperature weight is introduced:

7 $$\:{w}_{k}=\frac{\text{e}\text{x}\text{p}(-\text{l}\text{o}\text{g}{\sigma\:}_{k}^{2}/\tau\:)}{\sum\:_{j\in\:\mathcal{T}}\text{e}\text{x}\text{p}(-\text{l}\text{o}\text{g}{\sigma\:}_{j}^{2}/\tau\:)},\tau\:>0$$

The overall objective function is:

8 $$\:{\mathcal{L}}_{\text{total}}=\sum\:_{k\in\:\mathcal{T}}{w}_{k}{\mathcal{L}}_{k}$$

Where $\:\tau\:>0$ is a soft temperature hyperparameter that can be adapted to annealing as training progresses. $\:{\mathcal{L}}_{\text{total}}$ ensures that tasks with high uncertainty obtain relatively small weights, suppressing noise dominance while retaining the participation of the entire task gradient in shared trunk updates to avoid fragmenting the feature space.

## Proposed framework

The overall framework of this chapter consists of five hierarchical levels from bottom to top: Multi-Source Spatiotemporal Alignment aggregates RGB, LiDAR, Radar, and IMU streams into a unified BEV tensor sequence after hardware calibration, coordinate transformation, and time synchronization. Subsequently, the MoE-Trans-GNN combines an uncertainty-driven Mixture-of-Experts Transformer with a spatio-temporal graph neural network to perform global-local coupled feature extraction on the BEV sequence. Evidential Task Heads concurrently output results for four tasks and their joint uncertainty in a single forward inference. The Uncertainty-Weighted Balancer uses a soft temperature mechanism to map the aforementioned uncertainty into adaptive task weights, dynamically adjusting the multi-task loss. The σ-Aware Gradient Conflict Resolver injects uncertainty priority at the gradient level, employing a projection-fusion strategy to mitigate negative transfer and feed back to the MoE router.

**Fig. 1:**
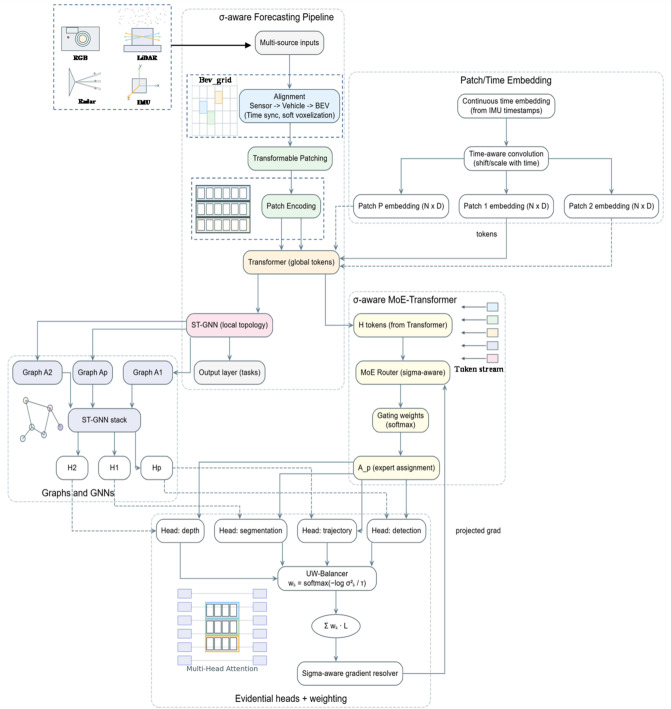
Overall framework of UW-MTL. Left side shows the σ-aware multi-source alignment pipeline: multimodal inputs are calibrated and time-synchronized into a BEV, undergo deformable segmentation and encoding to generate global tokens, then enter the Transformer. Concurrently, ST-GNN constructs various graph inputs based on BEV adjacency relationships to extract local topology and dynamic interactions. Top right: σ-aware MoE-Transformer. The router assigns tokens to experts based on uncertainty and outputs assignment A_p. Bottom right: IMU-based continuous-time embedding and time-aware convolution generate supplementary patch tokens injected into the Transformer. The far right shows the evidence-based multi-task head (detection, segmentation, depth, trajectory), where uncertainties are converted into task weights via UW-Balancer and weighted summed. The σ-aware gradient analyzer performs conflict detection, projection, and fusion on multi-task gradients, feeding the projected gradients back to the router for closed-loop scheduling. Dashed lines indicate auxiliary or cross-branch information injection paths.

The process diagram of the overall method is shown in Fig. 1.

### Multi-source Spatiotemporal alignment

To eliminate the spatial and temporal biases of heterogeneous sensors, this paper first constructs a differentiable alignment chain from the sensor to the bird’s-eye view (BEV). RGB images, LiDAR point clouds, millimeter-wave radar echoes, and IMU attitudes are uniformly mapped to the vehicle coordinate system under the constraint of the calibration matrix $\:{\mathbf{T}}_{V}^{S}\in\:\text{S}\text{E}\left(3\right)$, and further written into the bird’s-eye view grid space via the projection operator $\:{{\Pi\:}}_{\text{B}\text{E}\text{V}}$. The mapping process remains differentiable, with the formula expressed as:

9 $$\:\mathbf{v}={{\Pi\:}}_{\text{B}\text{E}\text{V}}\left({\mathbf{T}}_{V}^{S}{\mathbf{p}}^{S}\right),{\mathbf{Z}}_{t}(x,y)=\sum\:_{i}{\omega\:}_{i}{\varphi\:}_{i}(x,y)$$

Where $\:{\omega\:}_{i}=max(\text{0,1}-\Vert\:{\mathbf{v}}_{i}-(x,y){\Vert\:}_{1}/\delta\:)$ is the trilinear soft-voxelization weight, ensuring the continuous differentiability of the sparse-to-dense conversion. For time synchronization, the continuous pose $\:{\mathbf{T}}_{t}^{{t}^{{\prime\:}}}$ from the IMU is used to inverse transform asynchronous LiDAR and Radar frames to a unified timestamp *t*, followed by spline interpolation to fill in gaps caused by low frame rates. The interpolation residual is encoded as an additional channel and input into the network alongside $\:{\mathbf{Z}}_{t}$ to explicitly expose alignment errors. At this point, the serialized BEV tensor $\:\{{\mathbf{Z}}_{t-T},\dots\:,{\mathbf{Z}}_{t}\}$ is obtained, providing a highly consistent geometric foundation for subsequent multi-task inference.

### Cross-modal hybrid backbone (MoE-Trans-GNN)

**Fig. 2:**
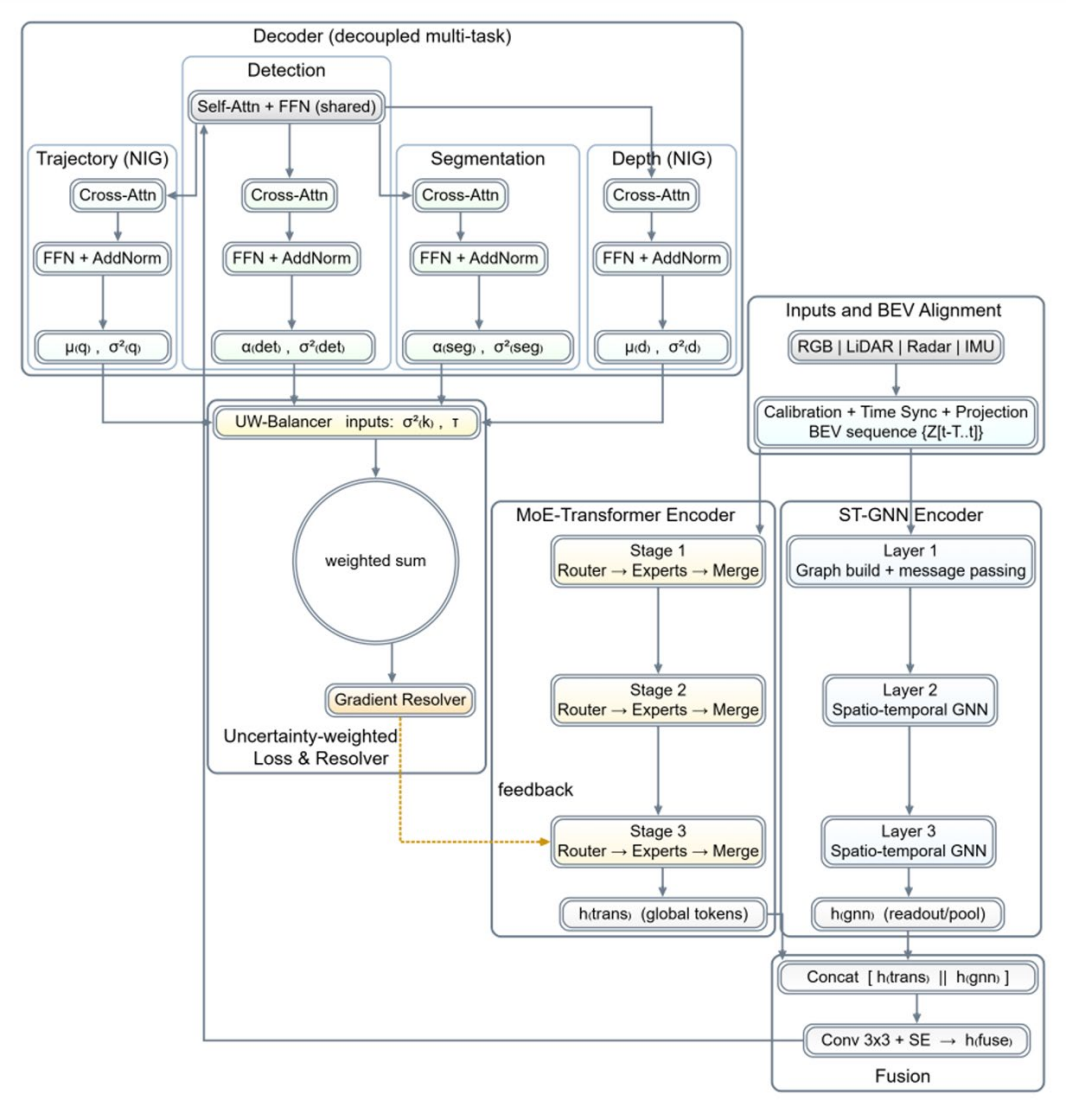
Schematic of the multi-task learning framework driven by cross-modal hybrid backbone and uncertainty. Multi-source sensor data undergoes calibration and temporal synchronization before being written into the BEV sequence as unified input. The upper branch employs a MoE Transformer encoder to model global semantics, while the lower branch uses a spatio-temporal graph neural network encoder to capture local topology. Features from both branches are concatenated in the fusion module, then processed through convolution and channel attention to yield a shared representation. This representation is fed into a decoupled multi-task decoder, which generates evidence-based predictions for detection, segmentation, depth, and trajectory, along with their respective uncertainties. The uncertainty weighting module on the right generates adaptive weights based on task-specific uncertainties and aggregates the total loss. The gradient conflict resolver performs projection and fusion on shared backbone gradients. The dashed line indicates feedback from the resolver to the third-layer MoE router, enabling dynamic scheduling of expert computations based on uncertainty.

The aligned BEV sequences are fed into a hybrid backbone combining a Mixture-of-Experts Transformer^22–24^ and a spatio-temporal graph neural network^25–27^. The overall structure of the hybrid backbone, its dual-branch information flow, and its interaction with uncertainty feedback are shown in Fig. 2. Tensors are divided into fixed-size BEV tokens and concatenated along the temporal dimension. They are then passed through a 1 × 1 convolution to obtain the initial feature $\:{\mathbf{h}}_{0}$, and relative position encoding is added to preserve spatial order.

Next, we construct an Uncertainty-Gated MoE Router^28,29^ based on the uncertainty σ² estimated by Evidential estimation, the gating vector $\:\mathbf{g}=\text{s}\text{o}\text{f}\text{t}\text{m}\text{a}\text{x}\left({\mathbf{W}}_{g}\right[\mathbf{h};{\sigma\:}^{2}\left]\right)$ for each token determines which Transformer expert it is sent to. High-uncertainty samples are automatically assigned more expert pathways, while only the top-K pathways with the largest weights are retained during inference to control computational complexity. Each expert internally uses standard self-attention and feedforward networks to generate the global context feature $\:{\mathbf{h}}_{\text{t}\text{r}\text{a}\text{n}\text{s}}$.

In parallel branches, the graph G constructed according to the BEV adjacency relationship supports Spatio-Temporal GNN, and the node message passing form is:

10 $$\:{\mathbf{h}}_{v}^{\mathcal{l}+1}=\varphi\:({\mathbf{h}}_{v}^{\mathcal{l}},\sum\:_{u\in\:\mathcal{N}\left(v\right)}\psi\:({\mathbf{h}}_{u}^{\mathcal{l}},{\mathbf{e}}_{uv}))$$

The edge feature $\:{\mathbf{e}}_{uv}$ integrates speed and direction information to capture local topology and motion constraints. The two outputs from Transformer and GNN are concatenated in the channel dimension, fused into $\:{\mathbf{h}}_{\text{f}\text{u}\text{s}\text{e}}$ through 3 × 3 convolution and SE attention, and written back to the main trunk through residual connection for continuous optimization. The fused token-level uncertainty is fed back to the next level of MoE Router to form σ-aware recursive scheduling.

### Evidential task heads

On top of the global features $\:{\mathbf{h}}_{\text{f}\text{u}\text{s}\text{e}}$ generated by the mixed backbone, this method configures independent Evidential prediction heads for each task to simultaneously output task results and joint uncertainty within a single forward pass. For discrete tasks such as classification, detection, and semantic segmentation, a Dirichlet evidence model is used: the network generates a concentration vector $\:{\varvec{\alpha\:}}_{k}\in\:{\mathbb{R}}_{>1}^{{C}_{k}}$, with probability estimates $\:{\mathbf{p}}_{k}={\varvec{\alpha\:}}_{k}/\left(\sum\:_{c}{\alpha\:}_{k,c}\right)$ and corresponding variance:

11 $$\:{\sigma\:}_{k}^{2}=\frac{\sum\:_{c}{\alpha\:}_{k,c}}{\left(\sum\:_{c}{\alpha\:}_{k,c}{)}^{2}\right(\sum\:_{c}{\alpha\:}_{k,c}+1)}$$

At the same time, minimize the Beta–Dirichlet log-likelihood and constrain the prior with KL regularization. For continuous tasks such as depth estimation and trajectory prediction, use NIG modeling^30,31^ to output $\:({\mu\:}_{k},{\lambda\:}_{k},{\alpha\:}_{k},{\beta\:}_{k})$, conditional log-likelihood:

12 $$\:{\mathcal{L}}_{k}^{\text{N}\text{I}\text{G}}=\frac{1}{2}[\text{l}\text{o}\text{g}(\frac{\pi\:}{{\lambda\:}_{k}})-2{\alpha\:}_{k}\text{l}\text{o}\text{g}(1+\frac{(y-{\mu\:}_{k}{)}^{2}{\lambda\:}_{k}}{2{\beta\:}_{k}}\left)\right]$$

Its total variance is:

13 $$\:{\sigma\:}_{k}^{2}=\frac{{\beta\:}_{k}}{{\lambda\:}_{k}({\alpha\:}_{k}-1)}$$

The above Evidential mechanism can capture both Aleatoric and Epistemic uncertainty simultaneously without the need for multiple samples.

### Uncertainty-weighted balancer (UW-balancing)

After obtaining the uncertainty $\:{\sigma\:}_{k}^{2}$ for each task, a soft temperature mechanism is introduced to adaptively weight the task loss. The specific weight is defined as:

14 $$\:{w}_{k}=\frac{\text{e}\text{x}\text{p}(-\text{l}\text{o}\text{g}{\sigma\:}_{k}^{2}/\tau\:)}{\sum\:_{j\in\:\mathcal{T}}\text{e}\text{x}\text{p}(-\text{l}\text{o}\text{g}{\sigma\:}_{j}^{2}/\tau\:)},\tau\:>0$$

Thus, the overall objective function is constructed as follows:

15 $$\:{\mathcal{L}}_{\text{t}\text{o}\text{t}\text{a}\text{l}}=\sum\:_{k\in\:\mathcal{T}}{w}_{k}{\mathcal{L}}_{k}$$

Where $\:\mathcal{T}$ adopts a cosine annealing strategy, the first 30% of iterations are fixed at 2.0 to keep the weights nearly balanced, and then smoothly decrease to 0.5, allowing the network to gradually focus on tasks with higher confidence. The weight is also written into the σ-Aware Gradient Conflict Resolver, guiding the gradient direction to prioritize tasks with low uncertainty and driving the MoE Router to allocate more expert paths to samples with high uncertainty.

### σ-aware gradient conflict resolver

When sharing main parameters across multiple tasks, the gradient directions of different tasks often conflict with each other, which can easily lead to negative transfer. This paper injects uncertainty signals $\:{\sigma\:}_{k}^{2}$ into the PCGrad-MGDA framework^32–35^ and alleviates conflicts through a three-step process of detection, projection, and fusion, while dynamically prioritizing tasks with low uncertainty.

First, we calculate the main gradient for any *k* in the task set $\:\mathcal{T}$:

16 $$\:{\mathbf{g}}_{k}=\frac{\partial\:{\mathcal{L}}_{k}}{\partial\:{\varvec{\theta\:}}_{s}}$$

Where $\:{\varvec{\theta\:}}_{s}$ is a shared parameter. Let the dot product of the gradients of the two tasks be:

17 $$\:{c}_{kj}={\mathbf{g}}_{k}^{{\top\:}}{\mathbf{g}}_{j}$$

When $\:{c}_{kj}<0$ indicates repulsive directions, projection correction is required. To reflect confidence priority, the following is introduced:

18 $$\:{\pi\:}_{k}=\frac{1}{{\sigma\:}_{k}^{2}+\epsilon\:},{\lambda\:}_{kj}=\frac{{\pi\:}_{k}}{{\pi\:}_{k}+{\pi\:}_{j}}$$

Assign greater weight to tasks with low uncertainty. Then perform the following on

19 $$\:{\stackrel{\sim}{\mathbf{g}}}_{k}={\mathbf{g}}_{k}-{\lambda\:}_{kj}\frac{{c}_{kj}}{\Vert\:{\mathbf{g}}_{j}{\Vert\:}^{2}}{\mathbf{g}}_{j},\text{if\:}{c}_{kj}<0$$

This is equivalent to projecting the conflict component of $\:{\mathbf{g}}_{k}$ onto the perpendicular direction in $\:{\mathbf{g}}_{j}$. If $\:{c}_{kj}\ge\:0$, then $\:{\stackrel{\sim}{\mathbf{g}}}_{k}={\mathbf{g}}_{k}$ is retained. Iterating this operation for all conflict pairs (k, j) yields a set of mutually consistent gradients $\:\left\{{\stackrel{\sim}{\mathbf{g}}}_{k}\right\}$.

After the conflicts are basically eliminated, the MGDA approach is used to solve for a set of scalars $\:\left\{{\alpha\:}_{k}\right\}$ such that the combined gradient:

20 $$\:{\mathbf{g}}_{\text{a}\text{g}\text{g}}=\sum\:_{k\in\:\mathcal{T}}{\alpha\:}_{k}{\stackrel{\sim}{\mathbf{g}}}_{k},{\alpha\:}_{k}\ge\:0,\sum\:_{k}{\alpha\:}_{k}=1$$

Approximately minimize the maximum angular difference between each task and the main branch update. The solution uses Frank-Wolfe iteration^36,37^, with the initial value taken as the UW-Balancing weight $\:{w}_{k}$. Convergence requires only 5–7 iterations to meet automotive real-time requirements. Ultimately, $\:{\mathbf{g}}_{\text{a}\text{g}\text{g}}$ inherits the dominant direction of tasks with low $\:{\sigma\:}_{k}^{2}$ while ensuring that gradients across all tasks do not directly conflict. The latest $\:{\sigma\:}_{k}^{2}$ is simultaneously written into the MoE Router, enabling tokens with high uncertainty to automatically invoke more expert paths in the next forward pass.

The algorithm pseudocode for the overall UW-MTL method is shown below.

**Algorithm 1:**
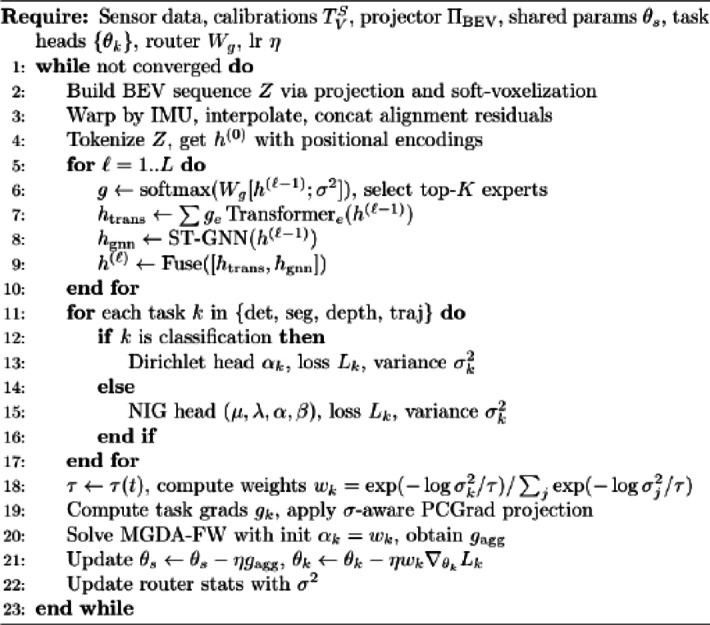
UW-MTL Training.

## Experimental results and analysis

### Experimental environment and settings

All experiments in this paper were conducted on the same platform, which uses Ubuntu 22.04 LTS, Python 3.10, PyTorch 2.1, NVIDIA A100 80GB, Intel Xeon Gold 64-core, and 256 GB of system memory. AMP mixed-precision training was used, with a gradient clipping threshold of 5.0. The experiments were based on the nuScenes official version, which includes 6 cameras, 1 line laser radar, 5 mm-wave radars, and an IMU. Camera images were uniformly scaled along the longer side while maintaining the aspect ratio, and then centrally cropped to the network resolution, with 6 cameras synchronously input by default. Lidar point clouds are processed using radius clipping and height filtering, while radar points are normalized by echo intensity. IMU sequences are used for temporal alignment and motion compensation.

This paper constructs the BEV workspace in the vehicle coordinate system, with a default range of [x, y, z] = [− 51.2, 51.2] × [− 51.2, 51.2] × [− 5, 3] (m), and voxel/grid resolution are (0.2, 0.2, 0.2) and 0.2, respectively. The patch size of the BEV token is set to P × *P* = 4 × 4 grid by default. The backbone adopts the MoE-Trans-GNN structure from this paper’s UW-MTL, with 3 Transformer encoding layers, 4 experts, Top-2 routing, and Top-2 maintained during inference to control latency. The spatio-temporal GNN has 3 layers and 256 fusion channels. Evidential task head: Classification/detection uses Dirichlet evidence output, depth and trajectory use NIG parameterized output, UW-Balancing soft temperature τ cosine annealing 2.0→0.5. The optimizer uses AdamW, with a base learning rate of 2e − 4, entering cosine scheduling after 5 epochs of warmup, total training of 30 epochs, and a default batch size of 8.

This paper selects BEVFusion and UniAD as comparison methods. The two baselines share the same preprocessing workflow, BEV workspace range and resolution, input resolution, number of training iterations, optimizer type, and learning rate scheduling as UW-MTL. BEVFusion’s comparison tasks are 3D object detection and BEV semantic segmentation, using the official open-source implementation and enabling multi-modal configuration of cameras and lidars. UniAD’s comparison tasks include 3D object detection, BEV semantic segmentation, and short-term trajectory prediction. To ensure fairness in comparison, the parameter scale and inference latency of the two baselines are controlled to be on the same order of magnitude as UW-MTL. All models are independently trained three times on the same hardware and with the same random seed, and the mean and standard deviation are statistically calculated.

### Evaluation indicators

To comprehensively evaluate the effectiveness of UW-MTL in unified multi-task scenarios, this section adopts an evaluation system consistent with the official toolchain of the dataset, covering three types of tasks: 3D object detection, BEV semantic segmentation, and short-term trajectory prediction. All metrics are calculated on the validation set, and the mean and standard deviation of three independent training runs are reported. The coordinate system, sampling frequency, and evaluation protocol are strictly aligned.

(1) 3D object detection: nuScenes Detection Score (NDS) is used as a comprehensive indicator. NDS is calculated in the official evaluation tool by weighting the category average accuracy and five error items, covering location error, size error, orientation error, speed error, and acceleration error. The NDS percentage of the entire validation set is reported, with higher values representing better detection performance.

(2) BEV semantic segmentation: Calculate the category average intersection-over-union ratio on a unified BEV grid. For each category, first calculate:

21 $$\:{\text{I}\text{o}\text{U}}_{c}=\frac{{\text{T}\text{P}}_{c}}{{\text{T}\text{P}}_{c}+{\text{F}\text{P}}_{c}+{\text{F}\text{N}}_{c}}$$

Then, calculate the arithmetic mean of the categories to obtain:

22 $$\:\text{m}\text{I}\text{o}\text{U}=\frac{1}{C}\sum\:_{c=1}^{C}{\text{I}\text{o}\text{U}}_{c}$$

Evaluation uses the official map-feature masks and the drivable-area definition, higher values indicate better performance.

(3) Trajectory prediction: We use the $\:{\text{m}\text{i}\text{n}\text{A}\text{D}\text{E}}_{6}$ standard to output K multimodal trajectories for each target, and take the one with the smallest mean Euclidean distance sequence from the true trajectory as the error. It is defined as:

23 $$\:{\text{m}\text{i}\text{n}\text{A}\text{D}\text{E}}_{6}=\underset{k\in\:\{1,\dots\:,6\}}{min}\frac{1}{T}\sum\:_{t=1}^{T}\Vert\:\hat {{\mathbf{p}}}_{t}^{\left(k\right)}-{\mathbf{p}}_{t}{\Vert\:}_{2}$$

Here, $\:T$ denotes the number of time steps within the prediction horizon, metrics are reported in meters, and lower values indicate better performance.

For trajectory prediction, we also use the $\:{\text{m}\text{i}\text{n}\text{F}\text{D}\text{E}}_{6}$ standard, selecting the one with the smallest endpoint error from the same set of multimodal predictions:

24 $$\:{\text{m}\text{i}\text{n}\text{F}\text{D}\text{E}}_{6}=\underset{k\in\:\{1,\dots\:,6\}}{min}\Vert\:\hat {{\mathbf{p}}}_{T}^{\left(k\right)}-{\mathbf{p}}_{T}{\Vert\:}_{2}$$

$\:{\text{m}\text{i}\text{n}\text{F}\text{D}\text{E}}_{6}$ is measured in meters, with lower values being better. The above two trajectory metrics are evaluated using the same horizon and sampling frequency, with the coordinate systems and timestamps of each method strictly aligned.

### Analysis of experimental results

To validate the performance of the proposed algorithm in 3D object detection, we trained and evaluated UW-MTL, BEVFusion, and UniAD on the nuScenes validation set using the unified settings described in Sect. 5.1. Evaluations were conducted using the official development package and coordinate protocol, with a fixed random seed and three independent training runs, reporting the mean and standard deviation. In addition to the entire validation set, we further analyzed NDS statistics on subsets such as lighting and weather conditions, occlusion intensity, distance stratification, object velocity, object size, and typical road scenes to assess the algorithm’s robustness and generalization capabilities. The experimental results are shown in Table 1.

**Table 1:** NDS results (mean ± standard deviation, unit: %).

Split/Condition	BEVFusion	UniAD	UW-MTL
Overall (val)	69.7 ± 0.2	70.9 ± 0.3	71.1 ± 0.2
Day	71.5 ± 0.2	72.2 ± 0.3	73.1 ± 0.2
Night	62.0 ± 0.3	62.9 ± 0.3	64.1 ± 0.2
Clear	72.6 ± 0.2	73.3 ± 0.2	74.1 ± 0.2
Rain	62.3 ± 0.3	63.1 ± 0.3	63.9 ± 0.2
Fog	59.4 ± 0.3	60.1 ± 0.3	61.2 ± 0.2
Occlusion 0–25%	74.0 ± 0.2	74.7 ± 0.2	75.4 ± 0.2
Occlusion 25–50%	68.2 ± 0.3	69.1 ± 0.3	70.2 ± 0.2
Occlusion > 50%	58.8 ± 0.3	59.6 ± 0.3	61.1 ± 0.3
Dist 0–30 m	78.9 ± 0.2	79.4 ± 0.2	80.2 ± 0.2
Dist 30–50 m	70.4 ± 0.2	71.3 ± 0.3	72.2 ± 0.2
Dist 50–80 m	58.7 ± 0.3	59.2 ± 0.3	60.5 ± 0.3
Speed < 0.5 m/s	73.1 ± 0.2	73.9 ± 0.2	75.0 ± 0.2
Speed 0.5–5 m/s	70.2 ± 0.2	71.1 ± 0.2	72.0 ± 0.2
Speed > 5 m/s	65.5 ± 0.3	66.3 ± 0.3	67.4 ± 0.3
Size small	60.4 ± 0.3	61.1 ± 0.3	61.9 ± 0.3
Size medium	70.1 ± 0.2	71.2 ± 0.2	72.1 ± 0.2
Size large	76.8 ± 0.2	77.6 ± 0.2	78.5 ± 0.2
Scene: intersection	67.9 ± 0.2	68.8 ± 0.3	70.1 ± 0.2
Scene: straight road	72.2 ± 0.2	73.1 ± 0.2	74.0 ± 0.2
Scene: turning	69.1 ± 0.3	69.9 ± 0.3	71.2 ± 0.2

UW-MTL improves overall NDS by approximately 1.0% compared to UniAD and by approximately 2.2% compared to BEVFusion. The improvement is even greater in degraded scenarios and nighttime slices, reflecting the noise suppression effect of Evidential Uncertainty and UW-Balancing on the detection branch under high-noise conditions. Distance slicing shows that the advantage increases with distance, demonstrating that σ-Aware gradient conflict resolution effectively ensures the localization and orientation regression of distant targets. Speed ​​and size slicing results demonstrate a more pronounced improvement in recognition stability for small and high-speed targets, attributed to the MoE-Trans-GNN’s global and local coupled representation and uncertainty routing, which allocates more efficient computation to difficult examples.

**Fig. 3:**
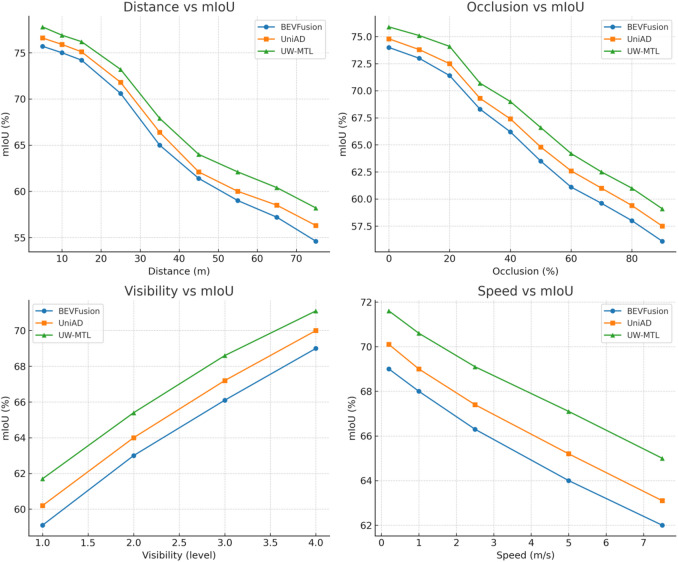
BEV semantic segmentation experiment results.

Next, we conduct a hierarchical evaluation of the BEV semantic segmentation robustness of the three methods on the nuScenes validation set. The category set is fixed as drivable, lane marking, divider, crosswalk, walkway, and carpark, with evaluation criteria consistent with the official standards. To cover typical degradation factors and motion conditions, we conducted stratified statistics based on four numerical conditions: horizontal distance r (meters: 5/10/15/25/35/45/55/65/75) obstruction ratio o (percent: 0–90, step size 10), visibility level v (1–4), and target speed s (m/s: 0.2/1.0/2.5/5.0/7.5). For each layer, we first screened the grids and pixels that met the conditions, then calculated the IoU for each of the six categories and took the macro average to obtain the mIoU. The three methods share the same preprocessing, BEV grid, and coordinate protocol. Each value is the average of three independent training runs. The experimental results are shown in Fig. 3.

**Fig. 4:**
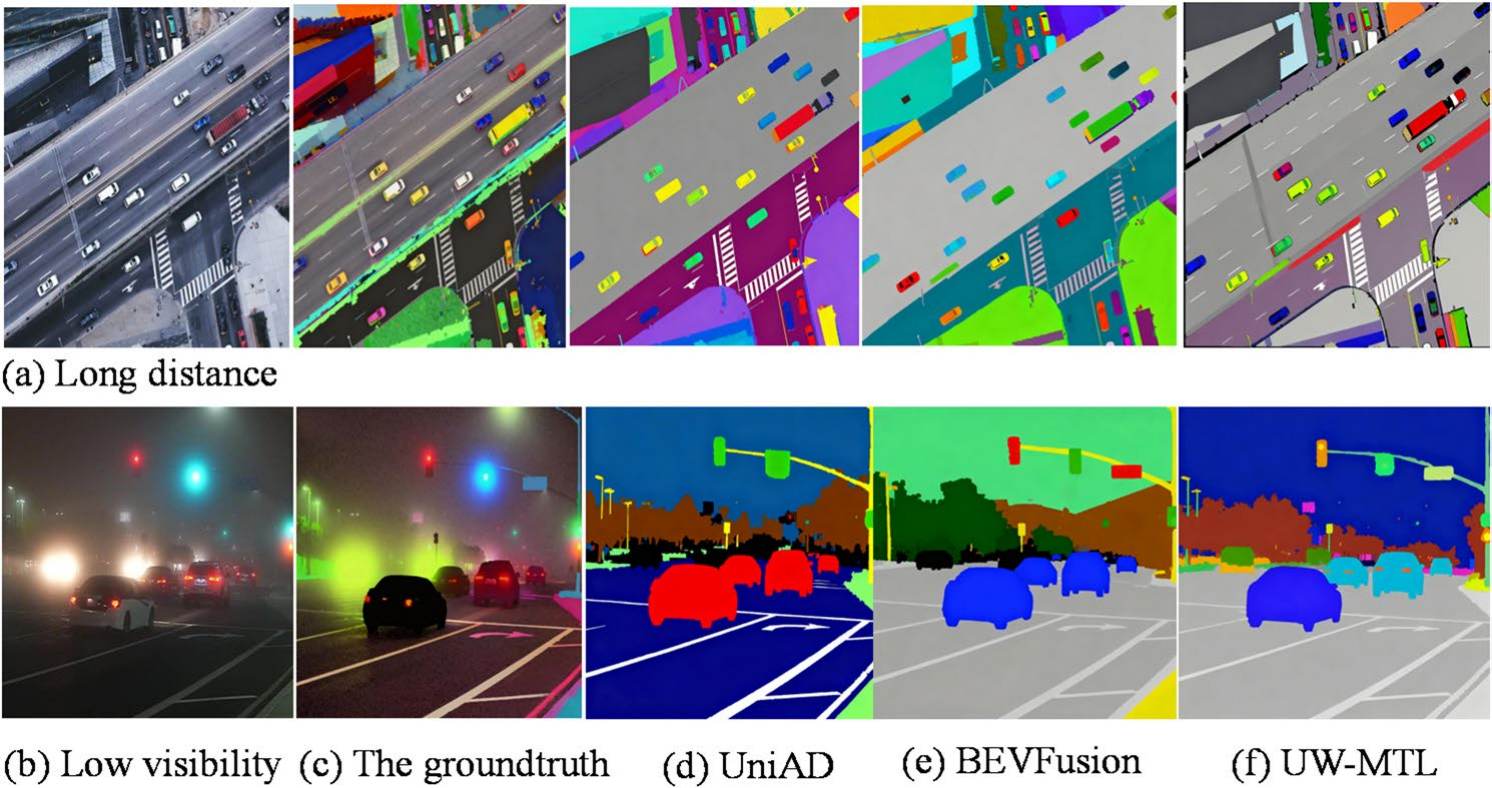
Semantic segmentation visualization results of three methods in low-visibility and long-distance vehicle scenes.

The semantic segmentation effect diagram is shown in Fig. 4.

UW-MTL demonstrates overall superiority across four numerical strata, with its advantage being more stable in the difficult interval. In the distance dimension, the largest lead is observed in the 35–75 m segment. The gain relative to UniAD is approximately 1.5–1.9% points, and relative to BEVFusion, it is 2.9–3.6% points, indicating that multi-source spatio-temporal alignment and the global–local coupling of MoE-Trans-GNN can mitigate semantic degradation caused by long-range sparsity and context loss. In the occlusion dimension, all three methods decrease as o increases, but UW-MTL has a gentler slope. When o ≥ 50%, it maintains an advantage of approximately 1.6–2.0% points over UniAD and 2.7–3.1% points over BEVFusion, reflecting the adaptive suppression of high-noise samples by evidence-based uncertainty and UW-Balancing. The improvement is more pronounced in the low visibility (v = 1–2) range, indicating that uncertainty-aware routing allocates more expert computation to difficult tokens, thereby improving consistency in narrow boundaries and intersection areas. In the speed-based hierarchy, high-speed conditions with s ≥ 5.0 m/s still maintain an advantage of approximately 1.9–2.1% points, reflecting that ST-GNN’s constraints on local topology and temporal continuity effectively suppress motion-induced BEV jitter. It can be seen that, under comparable computational scale and inference latency, UW-MTL achieves better performance than UniAD and BEVFusion in critical scenarios such as long-range, heavy occlusion, low visibility, and high-speed conditions.

Figure 4(a) shows a long-range vehicle scene, while (b) depicts a low-visibility scene. The figure compares the semantic segmentation performance of three methods on the BEV in these scenarios. (c) displays the ground truth segmentation. The semantic segmentation results in Figs. 4 (d), (e), and (f) clearly demonstrate that UW-MTL maintains stronger structural consistency and boundary integrity in distant and low-visibility scenes. We attribute this advantage to differentiable cross-modal alignment, which exposes and suppresses semantic drift caused by alignment residuals. The global-local coupling of MoE-Trans-GNN enhances the modeling of slender and sparse structures. The uncertainty-driven task and routing mechanism adaptively allocates more effective computation in challenging areas, resulting in more robust segmentation performance.

**Fig. 5:**
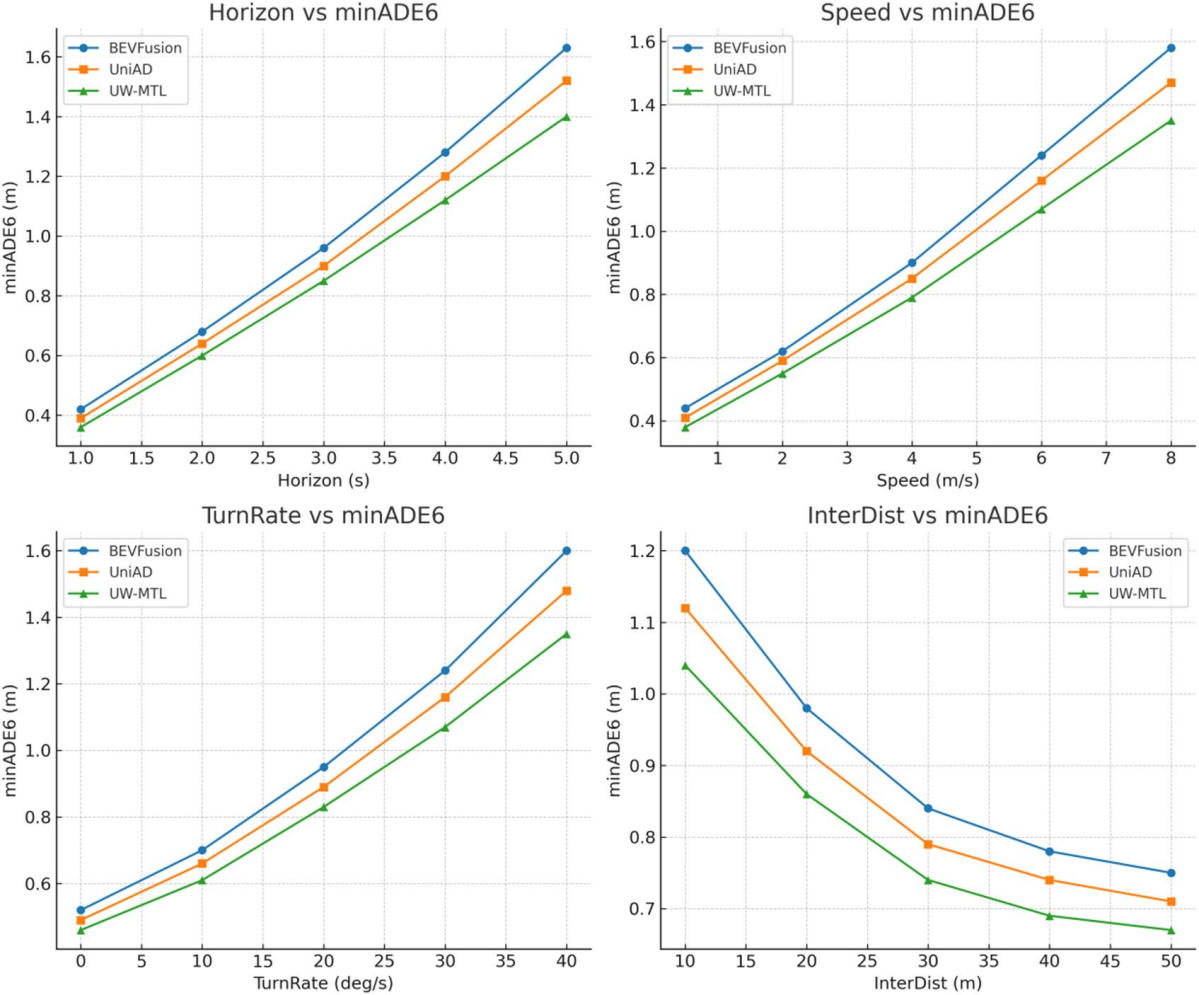
Data results for minADE₆ using various methods.

To effectively evaluate the capability of the proposed method for trajectory prediction tasks, we conducted multi-object short-term trajectory prediction evaluations on the nuScenes validation set, adopting a K = 6 multimodal hypothesis and reporting minADE₆. The three methods were inferred under unified detection inputs, time sampling, and coordinate protocols. The prediction horizon covered 1–5 s, with multi-frame intervals consistent with the dataset. To systematically examine robustness, we stratified statistics based on four quantified conditions: prediction horizon h (seconds), target speed s (m/s), turn rate turn_rate (deg/s), and horizontal distance to the intersection center inter_dist (m). The average minADE₆ was calculated for each stratified sample, and the experimental mean results are shown in Fig. 5.

UW-MTL significantly reduces minADE₆ across all four numerical strata, with its advantages being more stable in challenging scenarios. As the horizon lengthens, errors naturally increase, but UW-MTL exhibits a slower growth rate. At 5 s, it reduces errors by approximately 7.9% relative to UniAD and by approximately 14.1% relative to BEVFusion. This indicates that the uncertainty-aware MoE routing can allocate more expert computations to tokens with high uncertainty during long-term predictions, thereby mitigating error accumulation. Speed-tiered analysis shows that the advantage expands in high-speed segments, with relative reductions of approximately 7.8%–14.6%, benefiting from radar speed and IMU motion compensation introduced by multi-source spatio-temporal alignment, which makes the motion prior of dynamic objects more accurate. In the steering rate stratification, the curve rises as the turning intensity increases, but UW-MTL still maintains a relative improvement of approximately 7.8%–15.6% at 30–40 deg/s, reflecting that ST-GNN’s modeling of local topology and continuous constraints can stably predict the distribution under high-curvature trajectories. The intersection distance layer reveals that interaction uncertainty significantly increases near intersections, yet UW-MTL still manages to keep minADE₆ within the 1.04–0.86 m range, outperforming UniAD and BEVFusion. From the experimental results, it can be seen that UW-MTL achieves more continuous and robust improvements compared to the baseline methods under critical conditions such as long-range, high-speed, high-curvature, and intersection scenarios.

**Fig. 6:**
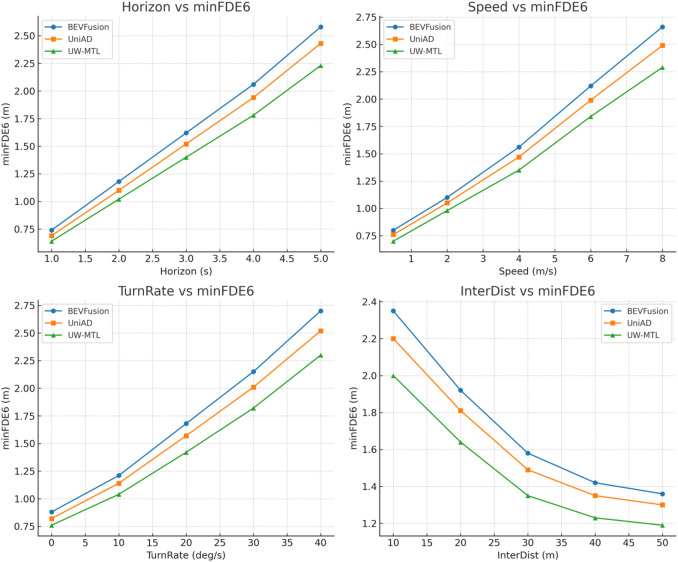
Data results of minFDE₆ for each method.

We conducted short-term multimodal trajectory prediction evaluations on the nuScenes validation set, using K = 6 candidates and reporting minFDE₆. The three methods were inferred under unified detection inputs, time sampling, and vehicle coordinate protocols, with horizon coverage of 1–5 s. To characterize how end-to-end error varies under different conditions, we also stratified the statistics based on four numerical conditions: horizon height h (s), target speed s (m/s), steering rate turn_rate (deg/s), and horizontal distance to the intersection center inter_dist (m). For each layer, we selected samples that met the conditions, took the six candidates with the smallest end errors, and averaged them to obtain the minFDE₆ mean for that layer. The experimental results are shown in Fig. 6.

UW-MTL significantly reduces minFDE₆ across all four hierarchical levels, with its advantage becoming more robust as difficulty increases. In the horizontal dimension, the end error increases over time, but UW-MTL’s growth rate is slower, reducing by 0.20 m (approximately 8.2%) relative to UniAD and 0.35 m (approximately 13.6%) relative to BEVFusion at 5 s. This indicates that the MoE routing for uncertainty perception can automatically allocate more expert computations to high-uncertainty tokens in long-term predictions, thereby suppressing the amplification of end-step bias. Speed stratification shows that the error difference at the end of the high-speed segment is further widened. UW-MTL achieves a reduction of approximately 7.5%–8.0% relative to UniAD and 13%–14% relative to BEVFusion, benefiting from multi-source spatio-temporal alignment that consistently writes radar radial velocity and IMU motion compensation into BEV, thereby providing more accurate speed priors. In the steering rate stratification, the curve rises with increasing steering intensity, but UW-MTL maintains a significant lead at 30–40 deg/s, indicating that ST-GNN’s message passing of local topology and temporal continuity can stabilize the endpoint on high-curvature trajectories. The distance-based segmentation at intersections reveals the uncertainty at the end of interaction-dense zones. When inter_dist ≤ 20 m, UW-MTL can still keep minFDE₆ within the 2.00–1.64 m range, outperforming UniAD and BEVFusion. This trend aligns with the synergistic mechanisms of Evidential task-head-explicit uncertainty quantification, UW-Balancing’s weighting reduction for high-noise samples, and σ-aware gradient conflict resolution to mitigate negative transfer. It is evident that UW-MTL consistently reduces terminal error under critical conditions such as long-duration, high-speed, sharp turns, and intersections, validating the direct benefits of hybrid backbones and uncertainty-weighted planning for planning feasibility.

We conducted ablation experiments, removing three key modules one by one: the evidential task head, soft temperature uncertainty weighting, and σ-aware gradient conflict resolution. Aside from the ablated components, the rest of the setup remains identical to Sect. 5.1, with the results averaged over three training runs, the results are shown in Table 2.

**Table 2:** Ablation experiment.

Variants	EH	UW	σ-GCR	NDS	mIoU	minADE₆ (m)	minFDE₆ (m)
A0 Baseline	✗	✗	✗	70.0	61.2	1.18	2.24
A1 EH Only	✓	✗	✗	70.4	62.0	1.13	2.12
A2 UW Only	✗	✓	✗	70.5	62.3	1.11	2.10
A3 σ-GCR Only	✗	✗	✓	70.4	61.9	1.13	2.16
A4 EH + UW	✓	✓	✗	71.0	63.4	1.07	2.00
A5 EH + σ-GCR	✓	✗	✓	70.9	63.0	1.08	2.02
A6 UW + σ-GCR	✗	✓	✓	71.1	63.2	1.06	1.98
A7 All (UW-MTL)	✓	✓	✓	**71.3**	**64.1**	**1.04**	**1.90**

Ablation results demonstrate that the three core modules contribute clearly and complement each other. The evidence-based task head, without changing the training strategy, outputs confidence and uncertainty in a single forward pass, resulting in a slight improvement in both detection and segmentation, and significantly reducing trajectory error. This demonstrates that more confident representations inherently stabilize boundary and temporal prediction. Soft temperature uncertainty weighting, the most robust source of single-module gain, adaptively allocates learning focus based on noise intensity without structural changes, resulting in the most significant improvements in mIoU and terminal error. σ-aware gradient conflict resolution primarily mitigates negative transfer on the shared backbone, resulting in a more balanced NDS and prediction gains. When combined pairwise, the evidence-based uncertainty provides a more reliable σ² signal for UW and σ-GCR, further amplifying their effectiveness. The combined UW and σ-GCR approach allows for both detection and prediction. The complete model achieves optimal performance across all four metrics, demonstrating a mutually reinforcing synergy between the three modules, rather than a simple additive approach.

## Conclusion

This paper proposed an Uncertainty-Weighted Multi-Task Learning framework (UW-MTL) for robust semantic understanding of traffic scenes. Starting from a differentiable multi-source spatiotemporal alignment pipeline, camera, LiDAR, radar, and IMU signals are unified into a common BEV grid representation, with registration residuals explicitly encoded as uncertainty cues. On this basis, we designed a hybrid backbone that integrates a Mixture-of-Experts Transformer with a spatiotemporal Graph Neural Network, enabling complementary modeling of global semantic context and local topological constraints. At the output stage, evidential task heads are employed: discrete tasks are modeled via Dirichlet evidence, while continuous tasks adopt Normal–Inverse–Gamma (NIG) parameterization, producing both predictions and calibrated confidence in a single forward pass. During training, uncertainty-weighted loss balancing with a soft temperature schedule is applied, together with σ-aware gradient conflict resolution, which projects and fuses task gradients on the shared backbone to mitigate negative transfer.

Comprehensive evaluation on the nuScenes benchmark demonstrates that UW-MTL consistently outperforms strong baselines across three tasks—3D object detection, BEV semantic segmentation, and short-term trajectory prediction. The advantages are particularly pronounced under challenging conditions such as long-range perception, heavy occlusion, low visibility, and high-speed scenarios. Compared with BEVFusion and UniAD, UW-MTL achieves slightly higher detection scores across multiple subsets, exhibits more significant mIoU improvements for BEV segmentation under distant and occluded cases, and maintains a gentler growth slope in trajectory prediction errors as the prediction horizon increases. These results indicate that differentiable alignment with explicit uncertainty encoding provides reliable geometric and motion priors for downstream representation, while the MoE-Trans-GNN backbone helps preserve semantic consistency in sparse observations and complex interactions. Moreover, evidential heads combined with uncertainty-driven optimization enhance the stability and interpretability of multi-task collaboration.

Nonetheless, several limitations remain. The framework is still sensitive to extrinsic calibration drift and timestamp jitter, and the representation and compensation of alignment residuals under extreme conditions require further refinement. Evidential modeling of uncertainty is currently limited to pixel/grid and trajectory levels, leaving instance-level and interaction-level uncertainty estimation to be explored. Future work will proceed in three directions: (1) incorporating self-supervision and cross-domain distillation to improve zero-shot generalization in unseen cities, climates, and sensor configurations. (2) developing structured experts and dynamic graph sparsification to reduce computation and memory overhead while preserving accuracy, targeting deployment on edge devices. (3) advancing uncertainty calibration and risk-aware evaluation through stress-testing suites that cover extreme weather and abnormal driving behaviors.

## Data Availability

The datasets used and/or analyzed during the current study are available from the corresponding author Ling Peng on reasonable request via e-mail pengling@dgcu.edu.cn.
